# A Closed-Loop Process for Rapid and Selective Lithium Extraction and Resynthesis from Spent LiFePO_4_ Batteries

**DOI:** 10.3390/molecules30122587

**Published:** 2025-06-13

**Authors:** Ruijing Liu, Yuxiao Liu, Jianjiang Li, Yuanlin Chen, Yule Zhu, Kunzheng Zhang, Shuxian Zhao, Liang Du, Xiaoyi Zhu, Lei Zhang

**Affiliations:** 1 College of Mechanical and Electrical Engineering, Qingdao University, Qingdao 266071, China; 13864223168@163.com (R.L.); jjli@qdu.edu.cn (J.L.); 13930175225@163.com (Y.Z.); 17861227940@163.com (K.Z.); 16716325866@163.com (S.Z.); 2 School of Environment and Geography, College of Environmental Science and Engineering, Qingdao University, Qingdao 266071, China; 3 Qingdao Grain & Oils Quality Inspection and Military Grain & Oils Supply Center, Qingdao 266042, China; chenyl4518@163.com; 4 Centre for Catalysis and Clean Energy, Gold Coast Campus, Griffith University, Southport, QLD 4222, Australia; liang.du@griffithuni.edu.au

**Keywords:** LiFePO_4_, spent lithium-ion batteries, leaching, recycling process, regeneration

## Abstract

The rapid growth of lithium iron phosphate (LiFePO_4_, LFP)-based lithium-ion batteries in energy storage raises urgent challenges for resource recovery and environmental protection. In this study, we propose a novel method for rapid and selective lithium extraction and the resynthesis of cathodes from spent LFP batteries, aiming to achieve an economically feasible and efficient recycling process. In this process, a selective leaching H_2_SO_4_-H_2_O_2_ system is employed to rapidly and selectively extract lithium, achieving a leaching efficiency of 98.72% within just 10 min. Through an exploration of the precipitation conditions of the lithium-containing solution, high-purity Li_2_CO_3_ is successfully obtained. The recovered FePO_4_ and Li_2_CO_3_ are then used to resynthesize LFP cathode materials through a carbon-thermal reduction method. A preliminary economic analysis reveals that the disposal cost of spent LFP batteries is approximately USD 2.63 per kilogram, while the value of regenerated LFP reaches USD 4.46, highlighting the economic advantages of this process. Furthermore, with an acid-to-lithium molar ratio of only 0.57—just slightly above the stoichiometric 0.5—the process requires minimal acid usage, offering clear environmental benefits. Overall, this work presents a green, efficient, and economically viable strategy for recycling spent LFP batteries, showcasing strong potential for industrial application and contributing significantly to the development of a circular lithium battery economy.

## 1. Introduction

In recent years, the rapid development of the electric vehicle industry and the large-scale deployment of renewable energy storage systems have led to explosive growth in the demand for lithium-ion batteries [1,2,3]. Among them, lithium iron phosphate (LFP) batteries have become the mainstream technology in the power battery sector due to their high safety, long cycle life, and low-cost advantages [4,5,6]. According to data from the China Automotive Battery Innovation Alliance, China’s total power battery production reached 778.2 GWh in 2023, with LFP batteries accounting for 531.4 GWh, representing a dominant market share of 68.29%. Market research predicts that the global LFP battery market will continue to expand at a compound annual growth rate of 6.39%, reaching USD 67.39 billion by 2029 [7]. However, as the first wave of commercial electric vehicles reaches their end-of-life cycle, it is estimated that 313,300 tons of spent LFP (SLFP) batteries will be generated globally by 2030. These retired batteries not only contain strategic resources such as lithium and phosphorus worth over CNY 20 billion but also pose environmental risks such as heavy metal pollution if improperly disposed [8,9,10]. Therefore, developing efficient and economical LFP battery recycling technologies has become a critical task for ensuring sustainable lithium supply and promoting the green development of the new energy industry [11].

Among the components of LFP batteries, the cathode material accounts for 36% of the total cost, making its efficient recycling crucial for both cost reduction and resource conservation [12,13]. Current cathode recycling technologies mainly revolve around three approaches: pyrometallurgy, hydrometallurgy, and direct regeneration [14,15,16,17,18]. Despite the simplicity of the pyrometallurgy process, it exhibits notable drawbacks including excessive energy consumption (exceeding 1000 °C), suboptimal metal recovery rates (Li < 50%), and a propensity to generate impurities such as LiFe(P_2_O_7_), Fe_x_P, and P_2_O_5_, as well as dioxins [19,20]. Direct regeneration can maintain the structural integrity of the material but requires stringent purity standards (impurity content < 3%), making it unsuitable for complex material systems in real-world recycling scenarios [21,22,23,24,25]. In contrast, hydrometallurgy has become the mainstream approach due to its high metal recovery rates (Li > 95%) and process controllability [26,27,28,29,30]. However, traditional hydrometallurgical processes still face two major technical bottlenecks. First, the need for excessive inorganic acid (the molar ratio of H_2_SO_4_/Li is 3.94 and the solid–liquid ratio is 100 g/L) results in significant wastewater generation. Secondly, the requirement for complex separation steps after the simultaneous leaching of iron and lithium significantly increases processing costs [31,32,33]. For example, Zheng et al. [34] achieved a 97.2% lithium leaching rate using a 2.5 mol L^−1^ H_2_SO_4_ system, but the process required 4 h of reaction time and generated a large amount of iron-containing wastewater. Zhang et al. [28] developed a sodium citrate-assisted leaching process that controlled the iron leaching rate at 5.1%, but it required 5 h of ball-milling pretreatment and an additive dosage 10 times the stoichiometric ratio, rendering it economically unfeasible.

**Figure 1:**
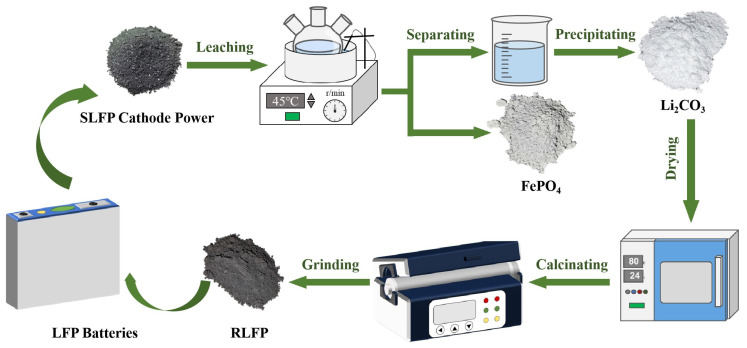
The short-process recycling system integrating “selective leaching, directional precipitation, and closed-loop regeneration”.

To address these technical challenges, this study innovatively constructed a short-process recycling system integrating “selective leaching, directional precipitation, and closed-loop regeneration” (Figure 1). Technical breakthroughs are reflected in the following aspects: First, a H_2_SO_4_-H_2_O_2_ collaborative leaching system was developed, which achieves the transformation of Fe^2+^ to Fe^3+^ by controlling the redox potential, simultaneously completing lithium leaching and iron–phosphorus fixation. This system enables selective lithium leaching (Li leaching rate > 98%, Fe leaching rate < 0.45%) within 10 min using near-stoichiometric acid, reducing reaction time by 95.8% and acid consumption by 85.53% compared to traditional processes [35,36]. Second, a pH gradient-controlled Li_2_CO_3_ directional precipitation model was established, which introduced a seed-induced growth mechanism to improve precipitation efficiency to 98.5% while controlling the mean particle size at 0.61 μm, making it directly usable for subsequent synthesis. Additionally, an innovative FePO_4_-Li_2_CO_3_ carbothermal reduction regeneration process was designed, and after 200 cycles at 0.1C, regenerated LFP maintained a discharge specific capacity of 128.9 mA h g^−1^, with a capacity retention rate of 90.77%. Economic evaluation shows that the cost of processing 1 kg of SLFP battery black powder is approximately USD 2.6255, a 41.2% reduction compared to traditional hydrometallurgical processes, while the process also decreases wastewater discharge by 76% [37,38]. This study provides a technically feasible and economically competitive solution for the high-value recycling of used LFP batteries, which contributes to the sustainable development of the lithium battery industry.

## 2. Results and Discussion

**Figure 2:**
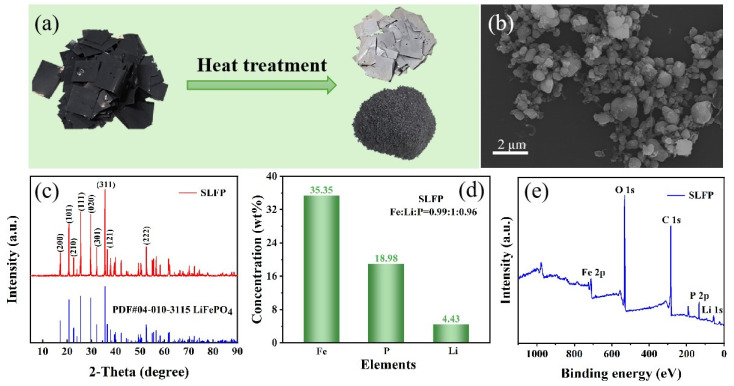
(**a**) Photographs of the SLFP cathode and the powder peeled off the aluminum foil, (**b**) the SEM images, (**c**) the XRD pattern, (**d**) the elemental content, and (**e**) the XPS spectra of SLFP cathode powder.

Figure 2a shows photographs of SLFP cathode powder before and after heat treatment. After heat treatment, the positive electrode material is easily peeled off the aluminum foil. The SEM image shows that the particles exhibit spherical morphology with a size of 0.17~1.31 μm (Figure 2b and Figure S1). The XRD pattern shown in Figure 2c displays diffraction angles at 35.591°, 25.561°, and 29.706° that correspond to the (311), (111), and (020) planes of crystalline LiFePO_4_ (PDF#04-010-3115), respectively. Figure 2d demonstrates that the elemental content of SLFP was 35.35% Fe, 4.43% Li, and 18.98% P. XPS was used to further study the surface element composition, and the Fe, O, C, P, and Li elements could be observed (Figure 2e).

The effect of different variables including the sulfuric acid concentration, H_2_SO_4_/Li molar ratio, leaching time, H_2_O_2_/Li molar ratio, and leaching temperature on the leaching rates of Li and Fe in SLFP was investigated.

**Figure 3:**
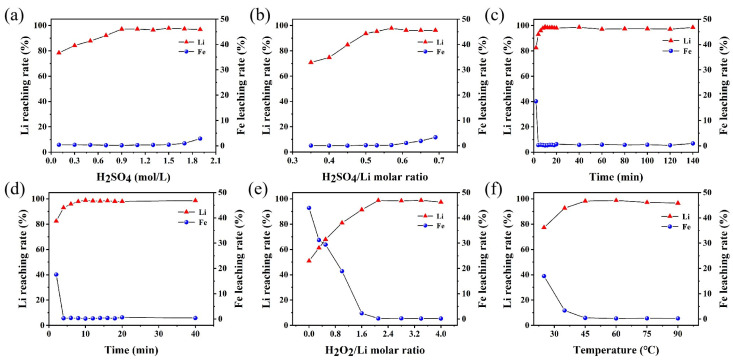
Influence of leaching conditions on leaching rates of Li and Fe. (**a**) Sulfuric acid concentration, (**b**) H_2_SO_4_/Li molar ratio, (**c**) leaching time, (**d**) leaching time within first 45 min, (**e**) H_2_O_2_/Li molar ratio, and (**f**) reaction temperature.

Sulfuric acid has been widely used as a leaching agent in the recycling of spent lithium-ion batteries. Traditional hydrometallurgical processes often employ excessive and high concentrations of H_2_SO_4_ to ensure complete metal leaching, but this method suffers from high reagent costs, complex subsequent processing, and significant wastewater discharge [39,40,41,42]. To find a local optimum within the considered experimental domain, the effect of H_2_SO_4_ concentration (0.1–1.9 M) on metal leaching behavior was systematically investigated under fixed conditions: H_2_SO_4_/Li molar ratio of 0.55, H_2_O_2_/Li molar ratio of 2.07, leaching temperature of 60 °C and time of 120 min, and stirring rate of 500 rpm. The experimental results (Figure 3a) show that Li exhibited highly selective leaching in the H_2_SO_4_ concentration range of 0.1–0.9 M, with its leaching efficiency significantly increasing from 78.38% to 97.13%, while the leaching efficiency of Fe remained below 0.5%. Notably, when the H_2_SO_4_ concentration exceeded 0.9 M, the leaching efficiency of Fe increased sharply from 0.21% to 2.86%, while the leaching efficiency of Li remained stable. Based on the selective leaching performance and economic considerations, 0.9 M was selected as the preferred H_2_SO_4_ concentration within the tested domain.

The mechanism of selective lithium extraction in the H_2_SO_4_-H_2_O_2_ system can be described by Equation (1):

2LiFePO_4_ + H_2_SO_4_ + H_2_O_2_ = Li_2_SO_4_ + 2FePO_4_↓ + 2H_2_O (1)

In this reaction, H_2_O_2_ acts as an oxidant to elevate the redox potential (ORP) of the solution, promoting the oxidation of Fe^2+^ to Fe^3+^. Under appropriate ORP conditions, Fe^3^⁺ rapidly reacts with PO_4_^3−^ to form insoluble FePO_4_, effectively suppressing Fe dissolution. Meanwhile, Li^+^ is released into the solution as Li_2_SO_4_. This ORP-driven selectivity ensures that Fe remains in the solid phase while Li is leached efficiently. The ORP value is optimized by adjusting the H_2_O_2_/Li molar ratio.

To investigate the effect of H_2_SO_4_ on the leaching rates of Li and Fe, experiments were conducted at 60 °C and 500 rpm using 0.9 M H_2_SO_4_, with a H_2_O_2_/Li molar ratio of 2.07 and a reaction time of 120 min. The experimental results are shown in Figure 3b. When the H_2_SO_4_/Li molar ratio increased from 0.35 to 0.57, the Li leaching rate significantly improved to 97.83%, while the Fe leaching rate remained below 0.3%. However, as the H_2_SO_4_/Li molar ratio further increased to 0.57–0.69, the Li leaching rate stabilized, whereas the Fe leaching rate continued to rise, reaching 3.31%. This indicates that excessive H_2_SO_4_ not only fails to enhance Li leaching but also dissolves some FePO_4_, leading to an increase in Fe leaching. Therefore, a H_2_SO_4_/Li molar ratio of 0.57 is appropriate.

Under the conditions of 0.9 M H_2_SO_4_, a H_2_SO_4_/Li molar ratio of 0.57, a H_2_O_2_/Li molar ratio of 2.07, a leaching temperature of 60 °C, and a stirring speed of 500 rpm, the effects of different reaction times ranging from 2 to 140 min on the leaching rates of Li and Fe were investigated. As shown in Figure 3c,d, after 2 min of reaction, the leaching rates of Li and Fe were 82.39% and 17.59%, respectively. With prolonged reaction time, the Li leaching rate gradually increased, reaching a peak of 98.90% at 10 min, while the Fe leaching rate decreased to 0.1621%. Further extension of the reaction time had minimal impact on the leaching rates of Li and Fe. Considering economic efficiency, the appropriate leaching time was determined to be 10 min.

Under the conditions of 0.9 M H_2_SO_4_, a H_2_SO_4_/Li molar ratio of 0.57, a leaching time of 10 min, a leaching temperature of 60 °C, and a stirring speed of 500 rpm, the effects of different H_2_O_2_/Li molar ratios on the leaching rates of Li and Fe were investigated. The results indicate that the reaction exhibits high selectivity upon the addition of H_2_O_2_ (Figure 3e). As the H_2_O_2_/Li molar ratio increases, the Li leaching rate rises rapidly and then stabilizes, while the Fe leaching rate shows the opposite trend. H_2_O_2_ acts as an oxidant, converting Fe^2+^ to Fe^3+^, thereby forming Li_2_SO_4_ and FePO_4_. When the H_2_O_2_/Li molar ratio reaches 2.1, the leaching rates of Li and Fe reach 98.72% and 0.219%, respectively, entering a stable phase. Further increasing the amount of H_2_O_2_ not only intensifies the reaction but also has limited effect on improving the Li leaching rate or reducing the Fe leaching rate. Therefore, a H_2_O_2_/Li molar ratio of 2.1 was selected for subsequent experiments.

The effect of temperature ranging from 25 °C to 90 °C on leaching efficiency was investigated under the conditions of 0.9 M H_2_SO_4_, a H_2_SO_4_/Li molar ratio of 0.57, a H_2_O_2_/Li molar ratio of 2.1, a leaching time of 10 min, and a stirring speed of 500 rpm. The experimental results (Figure 3f) show that at 25 °C, the Li leaching rate was 77.29%, while the Fe leaching rate reached 16.96%. Increasing the temperature significantly enhanced the molecular energy of the reactants and reduced the activation energy, thereby accelerating the reaction process of Equation (1). Meanwhile, as the dissolution process of FePO_4_ in water is an exothermic reaction, the solubility of FePO_4_ decreases as the temperature rises, which further reduces the leaching of iron. At 45 °C, the Li leaching rate increased to 98.27%, while the Fe leaching rate decreased to 0.4306%. Further increasing the temperature resulted in a slight improvement in the Li leaching rate and a minor decrease in the Fe leaching rate, but the changes were limited. Considering both the leaching efficiency and energy consumption, 45 °C was determined to be the appropriate reaction temperature.

Therefore, the local optimal conditions based on the OVAT method are as follows: the concentration of H_2_SO_4_ is 0.9 M, the molar ratio of H_2_SO_4_/Li is 0.57, the molar ratio of H_2_O_2_/Li is 2.1, the leaching time is 10 min, the temperature is 45 °C, and the stirring speed is 500 rpm. At this time, the solid–liquid ratio is 186.9 g/L, which is much higher than the 100 g/L of the traditional process [34]. We collated the relevant literature and made comparisons in terms of the leaching rate, leaching conditions, products, advantages and limitations, etc. For details, please refer to Table S1.

After purification and concentration, the leaching solution was treated with Na_2_CO_3_ to precipitate and recover Li in the form of Li_2_CO_3_. The leaching solution was obtained under local optimal conditions (0.9 M H_2_SO_4_, H_2_SO_4_/Li molar ratio of 0.57, H_2_O_2_/Li molar ratio of 2.1, leaching time of 10 min, temperature of 45 °C, and stirring speed of 500 rpm), and the impurities were removed by adjusting the pH with a NaOH solution. Then the obtained solution was concentrated to different concentrations, and the subsequent precipitation experiment was carried out at 95 °C for 15 min.

Figure S2a–e show the influence of Na_2_CO_3_ addition on the Li precipitation rate at different Li concentrations. When the Li concentration was 2 g L^−1^, no Li_2_CO_3_ precipitation was observed. When the concentration of Li concentration increased to 10 g L^−1^, with the addition of stoichiometric Na_2_CO_3_, the precipitation rate of Li was 59.21% (Equation (2)). When the concentration was further increased to 25 g L^−1^, the stoichiometric Na_2_CO_3_ addition achieved a Li precipitation rate of 90.77%. Excess Na_2_CO_3_ did not significantly improve the precipitation rate, so the appropriate conditions were determined to be concentrating the leaching solution to 25 g L^−1^.

Li_2_SO_4_ + Na_2_CO_3_ = Li_2_CO_3_↓ + Na_2_SO_4_ (2)

The XRD pattern (Figure S3) indicates that the obtained Li_2_CO_3_ matches well with the standard card (PDF#97-010-0324), and no visible characteristic peaks of other phases are observed, indicating that the lithium of SLFP can be recycled as Li_2_CO_3_. Figure S4 shows the photo and microstructure of the recovered Li_2_CO_3_. It presents white crystals and a layered stacking structure with a size of 0.38~1.22 μm.

**Figure 4:**
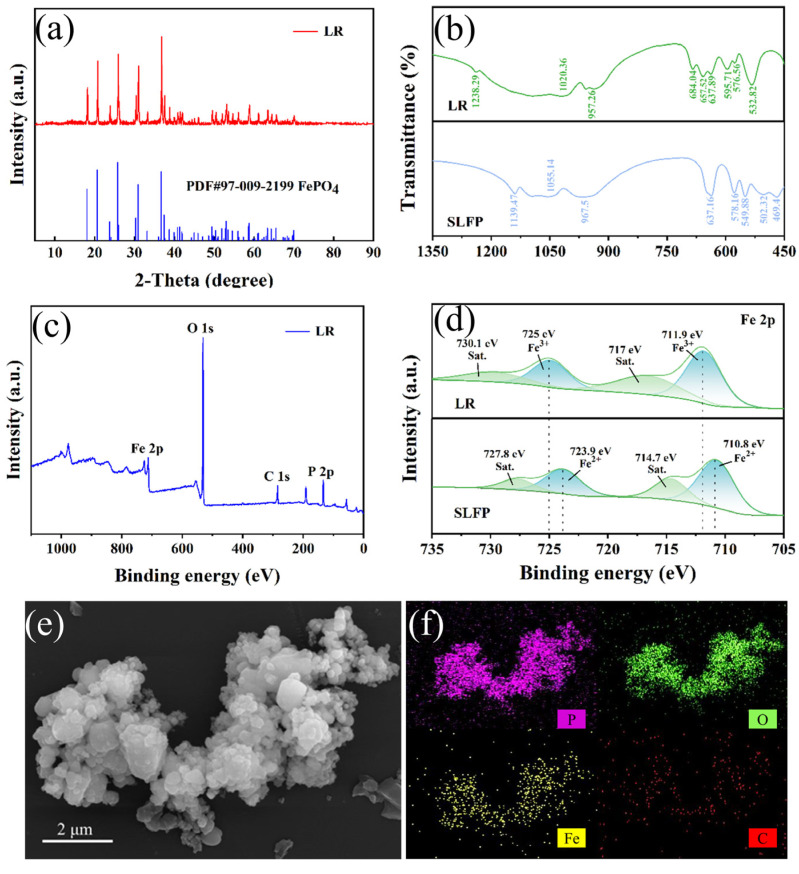
(**a**) XRD pattern of LR, (**b**) FT-IR spectra of LR and SLFP, (**c**) XPS spectra of LR, (**d**) Fe 2p spectra of LR and SLFP, (**e**) SEM image of LR, and (**f**) elemental mapping of LR.

To study the reaction mechanism for the recovery of Li from SLFP via a selective leaching H_2_SO_4_-H_2_O_2_ system and H_2_O_2_, a series of characterization studies of the products from the leaching process was carried out. The XRD pattern of the leaching residue (LR) is in good agreement with the standard card (PDF#97-009-2199), and no other peaks are found, indicating that Fe is recovered in the form of high-purity FePO_4_ (Figure 4a).

The FT-IR spectrum of the LR is shown in Figure 4b. The characteristic peak band of the FePO_4_ olivine structure was clearly observed at 1238.29 cm^−1^ [43]. This change in the valence state of Fe during the leaching process led to a slight shift in the vibration frequencies of the FT-IR spectrum. The stretching vibration bands corresponding to PO_4_^3−^ shifted from 967.5 cm^−1^ and 1055.14 cm^−1^ to 957.26 cm^−1^ and 1020.36 cm^−1^, respectively. The stretching vibration bands corresponding to octahedral FeO_6_ shifted to 657.52 and 684.04 cm^−1^ [44]. The peaks corresponding to the PO_4_ tetrahedron moved from 578.16 cm^−1^ and 549.88 cm^−1^ to 576.56 cm^−1^ and 532.82 cm^−1^ [45]. Additionally, the peak bands at 502.32 cm^−1^ and 469.4 cm^−1^ were related to the movement of Li^+^, and their intensities weakened as Li^+^ was stripped from SLFP [46].

XPS was used to study the valence state changes in elemental iron during the leaching process (Figure 4c). As shown in Figure 4d, the characteristic Fe^2+^ peaks in the SLFP appeared at 710.8 eV and 723.9 eV in the Fe 2p_3/2_ and Fe 2p_1/2_ regions, respectively. In the XPS spectrum of the LR, the peaks at 711.9 eV and 725 eV in the 2p_3/2_ and 2p_1/2_ regions, respectively, were the characteristic peaks of Fe^3+^, and no Fe^2+^ peaks were detected. This indicates that almost all the Fe^2+^ in SLFP was oxidized to Fe^3+^ in the LR. Compared with SLFP, the P 2p high-resolution XPS spectrum of the LR showed no significant change (Figure S5). The C 1S spectrum of the LR (Figure S6) revealed the characteristic peak of C=O/O–C=O, which indicates that a certain amount of carbon had undergone oxidation.

Figure 4e shows the SEM image of the LR. The particle size of the LR (Figure S7) is smaller than that of SLFP due to Li release, and the spherical particle size is relatively homogeneous. The characterization tests indicate that SLFP is directly converted to the olivine structure of FePO_4_ via an in situ reaction. The corresponding element mapping image (Figure 4f) shows that the Fe, P, and O elements are evenly distributed throughout the selected area. In addition, a small amount of the element C is detected, which may be derived from the remaining conductive agent in the LFP electrode residue.

**Figure 5:**
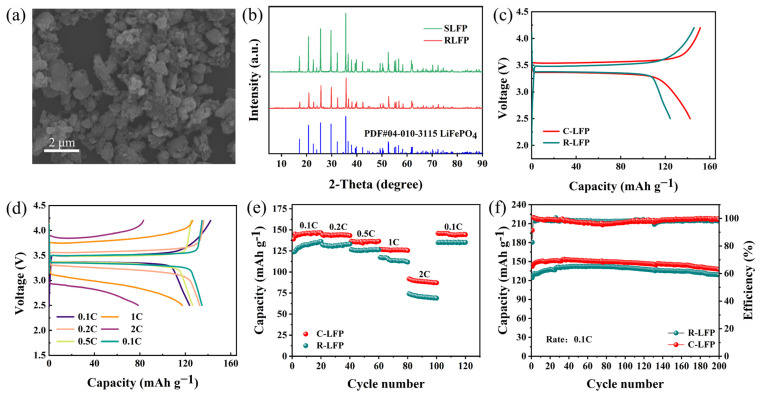
(**a**) SEM image of RLFP, (**b**) XRD patterns of RLFP and SLFP, (**c**) initial charge–discharge profiles of RLFP and CLFP at 0.1C, (**d**) initial charge–discharge curves of RLFP at different rates, (**e**) rate capability of RLFP and CLFP, and (**f**) cycling performance of RLFP and CLFP at 0.1C.

Figure 5a shows the SEM image of regenerated lithium iron phosphate (RLFP), exhibiting spherical morphology with a size of 0.19~1.23 μm (Figure S8), similar to commercial lithium iron phosphate (CLFP) (Figure S9). The smaller size is conducive to enhancing the mobility of Li^+^, reducing the interface resistance and polarization of the electrode electrolyte, and thus improving electrochemical performance. The XRD patterns of SLFP and RLFP are shown in Figure 5b. The peak intensity of RLFP is weaker than that of SLFP, which indicates that there is a defect in the crystallinity of RLFP. It is consistent with the standard LFP card (PDF#04-010-3115), and no impurities were detected.

To further demonstrate the feasibility of the closed-loop process, half cells of R-LFP and CLFP were assembled separately, and their electrochemical properties were tested. As shown in Figure 5c, the first discharge capacity of RLFP is 124.5 mA h g^−1^, slightly lower than that of CLFP (142.3 mA h g^−1^).

At rates of 0.1C, 0.2C, 0.5C, 1C, and 2C, the initial discharge specific capacities of RLFP are 123.6, 132.6, 126.5, 117.4, and 74.1 mA h g^−1^, respectively, lower than those of CLFP (139.1, 144.1, 136.4, 127, and 91.8 mA h g^−1^) (Figure 5d,e), which may be related to trace impurities in the recycled material. At a rate of 0.1C, the discharge capacity of RLFP reaches 92.96% of that of CLFP after the 20th cycle. However, at a 2C rate, the capacity of RLFP after the 20th cycle is only 79.10% of that of CLFP, indicating that capacity decay occurs more rapidly under high-current conditions. When the rate is reduced to 0.1C, the capacity of RLFP recovers to 134.8 mA h g^−1^, confirming good electrochemical reversibility and structural stability.

More importantly, after 200 cycles at 0.1C, RLFP maintained a discharge specific capacity of 128.9 mA h g^−1^, with a capacity retention rate of 90.77%; the material maintains relatively stable capacity during cycling (Figure 5f). Overall, the short-process recycling process collects high-purity Li_2_CO_3_ and uses the leaching products to regenerate a LiFePO_4_ cathode with good electrochemical properties, forming a sustainable closed loop.

Based on the experimental data in Figure 3 and Table 1, we conducted a preliminary assessment of the costs associated with the developed process. The cost references for chemicals (such as H_2_SO_4_ and H_2_O_2_), electricity, etc., are shown in Table S2. The cost of processing 1 kg of SLFP battery black powder is approximately USD 2.6255, while the value of the RLFP product is USD 4.4550, contributing the majority of the profit (Table S3). The product value is sufficient to cover the costs of chemicals and electricity. Furthermore, with the potential rebound in LFP prices and the cost reductions achievable through scaling up production, profits are expected to increase further. The process has the characteristics of low chemical and energy consumption, simplicity, the good electrochemical performance of the product, and the amount of acid used being basically less than in others, demonstrating that it is not only in line with the concept of green production but also has the advantage of environmental protection, which shows the great potential of SLFP battery recycling. Future work should focus on a more precise evaluation of economic and environmental benefits based on pilot-scale laboratory tests, including considerations of labor costs, equipment maintenance, and waste treatment expenses.

## 3. Experimental Section

### 3.1. Materials and Pretreatment

The spent LFP batteries used in this study were supplied by EVE Energy Co., Ltd. (Huizhou, China). All chemical reagents, including H_2_SO_4_, H_2_O_2_, Na_2_CO_3_, NaOH, and glucose, were of analytical grade and obtained from Sinopharm Chemical Reagent Co., Ltd. (Shanghai, China). The solutions used in this experiment were prepared with ultrapure water (resistivity: 18.2 MΩ·cm, UPT-II-20T, Ulupure Technology Co., Ltd., Chengdu, China).

The positive electrode pieces were first cut into small pieces, and then the small pieces were placed in a tube furnace and heat-treated at 500 °C in an argon atmosphere for 30 min. Finally, the waste LFP cathode powder was obtained by peeling off the aluminum foil. The cathode powder was treated using the wet acid digestion method. First, 0.1 g powder was added to a beaker, followed by an appropriate amount of concentrated hydrochloric acid, and then reacted at 70 °C for a specified duration. After digestion, a clarified solution was obtained, which was then diluted and used for further analysis.

### 3.2. Instrumentation and Analysis Method

The elemental content in the solution was determined using an inductively coupled plasma optical emission spectrometer (ICP-OES, Agilent 5800, Agilent Technologies Inc., Santa Clara, CA, USA); both Li and Fe adopted the radial view mode. The composition and structure of the samples were analyzed using an X-ray diffraction (XRD) instrument (Smart Lab 3KW, Rigaku Corporation, Akishima, Japan); the morphology of the solid particles was observed using a scanning electron microscope (SEM, JSM-6390LV, Japan Electronics Co., Ltd., Akishima, Japan); and the distribution of elements in the products was analyzed using an energy-dispersive spectrometer (EDS, X-Max^N^ 80T, Oxford Instruments, Abingdon-on-Thames, UK). Changes in the chemical valence states of the elements during leaching were studied using an X-ray photoelectron spectroscopy analyzer (XPS, K-Alpha X, Thermo Fisher Scientific, Waltham, MA, USA). The molecular structure was identified by a Fourier-transform infrared spectrometer (FTIR, IRTracer-100, Shimadzu Corporation, Kyoto, Japan).

### 3.3. Leaching and Resynthesis Processes

The leaching parameters were studied using the One Variable at a Time (OVAT) method. This means that what is found within the considered experimental domain is the local optimal value rather than the true global optimal value. The leaching of the cathode material is typically conducted in a 250 mL three-necked flask. First, 3.16 g cathode material was mixed with varying amounts of H_2_SO_4_ and H_2_O_2_, with a constant stirring speed of 500 rpm maintained throughout the leaching experiments. After the reaction was complete, the mixture was filtered. The filtrate was then diluted to an appropriate concentration before being analyzed using ICP-OES. The leaching rate of each element was determined using the following Equation (3):

(3) $$X_{\theta }=\frac{C_{\theta }\times V}{m\times W_{\theta }}\times 100\%$$

In the equation, C_θ_ (g/L) and V (L) represent the concentration of element θ in the filtrate and the volume of the filtrate solution, respectively. m (g) and W_θ_ represent the mass of the cathode material and the mass fraction of element θ in the cathode material, respectively. The filtrate obtained after leaching was used to adjust the pH with 10 wt% NaOH solution to precipitate trace amounts of iron and aluminum, and then it was evaporated and concentrated to obtain a solution with a specific lithium-ion concentration. At 95 °C, Na_2_CO_3_ was introduced and allowed to react for a designated period, resulting in the formation of a white precipitate. The mixture was filtered, and the filter residue was washed with boiling ultrapure water, dried to constant weight, and ground to yield Li_2_CO_3_. The post-leaching filter residue was dried and calcined in a tubular furnace at 600 °C under an air atmosphere for 4 h to remove residual carbon, followed by grinding to produce FePO_4_. Finally, LiFePO_4_/C was synthesized via a carbothermal reduction method using the recovered Li_2_CO_3_ and FePO_4_ as raw materials in a Li:Fe:P molar ratio of 1.05:1:1. Glucose (20 wt%) served as the carbon source to reduce Fe^3+^ to Fe^2+^, while simultaneously forming a continuous carbon network through thermal decomposition, thereby enhancing electrical conductivity.

## 4. Conclusions

This study developed a closed-loop process for the rapid and selective extraction of lithium from SLFP batteries, followed by resynthesis, demonstrating both economic viability and high efficiency. The process employs a selective leaching H_2_SO_4_-H_2_O_2_ system, achieving highly selective lithium leaching (with a leaching rate of 98.72%) and efficient recovery of FePO_4_, while controlling the iron leaching rate at just 0.219%. Through an exploration of the precipitation conditions of the lithium-containing solution, high-purity Li_2_CO_3_ was obtained, which was then combined with FePO_4_ through a carbothermal reduction process to synthesize a new LFP cathode. Preliminary economic assessments indicate that the cost of processing one kilogram of SLFP battery black powder is approximately USD 2.6255, while the value of RLFP reaches USD 4.455, highlighting significant economic benefits. In addition, very little acid is used in the process, indicating an advantage in terms of environmental protection.

Although the OVAT method effectively reduces the actual operation parameters, it is ultimately not globally optimal. Future optimizations based on Design of Experiments (DoE) can further improve this process. Although the process shows promising potential at the laboratory scale, further optimization is needed to address challenges in industrial-scale application, including the precise control of reaction conditions, equipment costs, and energy consumption in large-scale production. Moreover, the preliminary economic assessment did not account for labor, equipment maintenance, or waste treatment costs, necessitating a more comprehensive analysis.

## Figures and Tables

**Table 1:** Experimental input, energy consumption, and waste per batch (one batch is 1.0 kg black powder).

Materials	Amount per Batch	Cost Per Ton (USD) Market Price in China in 2024	Cost Per Batch (USD)
Waste LFP battery black powder (kg)	1.0	1390.1	1.3901
H_2_SO_4_ (L)	0.196	37.14	0.0073
Leaching water (L)	5.154	0.41	0.0021
Washing water (L)	3.165	0.41	0.0013
H_2_O_2_ (L)	1.343	112.90	0.1516
NaOH (kg)	0.032	372.33	0.0119
Na_2_CO_3_ (kg)	0.332	273.51	0.0908
Glucose (kg)	0.227	345.18	0.0784
N_2_ (L)	5.400	61.85	0.3340
Electricity		0.107/KW h	0.5580
Leaching–heating/stirring (KW h)	0.222		
Leaching–filtration/drying (KW h)	0.650		
Calcination of filter residue (KW h)	0.410		
Precipitation–evaporation (KW h)	0.143		
Precipitation–drying (KW h)	0.650		
Ball milling (KW h)	1.380		
Synthetic calcination (KW h)	1.760		
Total electricity calculation (KW h)	5.215		
Total			2.6255

## Data Availability

Data are contained within the article and Supplementary Materials.

## Supplementary Materials

The following supporting information can be downloaded at: https://www.mdpi.com/article/10.3390/molecules30122587/s1, Figure S1. The particle size distribution of SLFP; Figure S2. The influence of Na_2_CO_3_ addition on the Li precipitation rate at different Li concentrations: (a) 2g/L, (b) 10g/L, (c) 15g/L, (d) 20g/L, (e) 25g/L; Figure S3. XRD pattern of the obtained Li_2_CO_3_ precipitation recovered from SLFP; Figure S4. The photo (a), SEM image (b) and particle size distribution (c) of the recovered Li_2_CO_3_ precipitation recovered from SLFP; Figure S5. XPS spectra of P 2p: (a) SLFP, (b) LR; Figure S6. XPS spectra of C 1s: (c) SLFP, (d) LR; Figure S7. The particle size distribution of LR; Figure S8. The particle size distribution of RLFP; Figure S9. The SEM image (a) and the particle size distribution (b) of CLFP; Table S1. Comparison of the relevant literature on the selective leaching of LFP cathode materials; Table S2. Reference sources for parameter costs; Table S3. Cost and net product value for one batch experiment. References [47,48,49,50,51,52] are cited in the supplementary materials.
