# Supplementary material for: A Closed-Loop Process for Rapid and Selective Lithium Extraction and Resynthesis from Spent LiFePO4 Batteries
Source: Molecules. 2025 Jun 13;30(12):2587. doi: 10.3390/molecules30122587 (PMC12196130; doi:10.3390/molecules30122587)
Supplement: Supplementary file 1 [file molecules-30-02587-s001.zip › molecules-3633823-supplementary.pdf]

A closed-loop process for rapid and selective lithium extraction and resynthesis from spent $LiFePO_4$ batteries

Ruijing Liu [1,†], Yuxiao Liu [1,†], Jianjiang Li [1], Yuanlin Chen [2], Yule Zhu [1], Kunzheng Zhang [1], Shuxian Zhao [1], Liang Du [3], Xiaoyi Zhu [1,*] and Lei Zhang [3,*]

[1] College of Mechanical and Electrical Engineering, College of Environmental Science and Engineering, Qingdao University, Qingdao 266071, China; 13864223168@163.com (R.L.); liuyuxiao@qdu.edu.cn (Y.L.); jjli@qdu.edu.cn (J.L.); 13930175225@163.com (Y.Z.); 17861227940@163.com (K.Z.); 16716325866@163.com (S.Z.)

[2] Qingdao Grain&Oils Quality Inspection and Military Grain&Oils Supply Center, Qingdao 266042, China; chenyl4518@163.com

[3] Centre for Catalysis and Clean Energy, Gold Coast Campus, Griffith University, Southport, QLD 4222, Australia; liang.du@griffithuni.edu.au

* Correspondence: xyzhu@qdu.edu.cn (X.Z.); lei.zhang@griffith.edu.au (L.Z.)

† These authors contributed equally to the work.

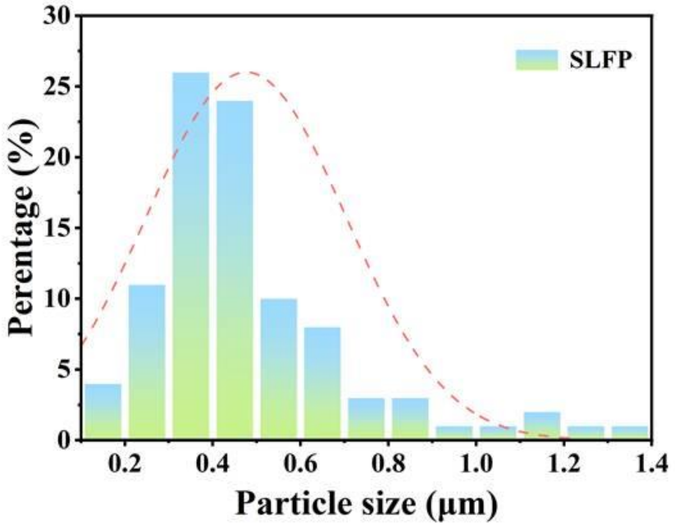

Figure S1. The particle size distribution of SLFP.

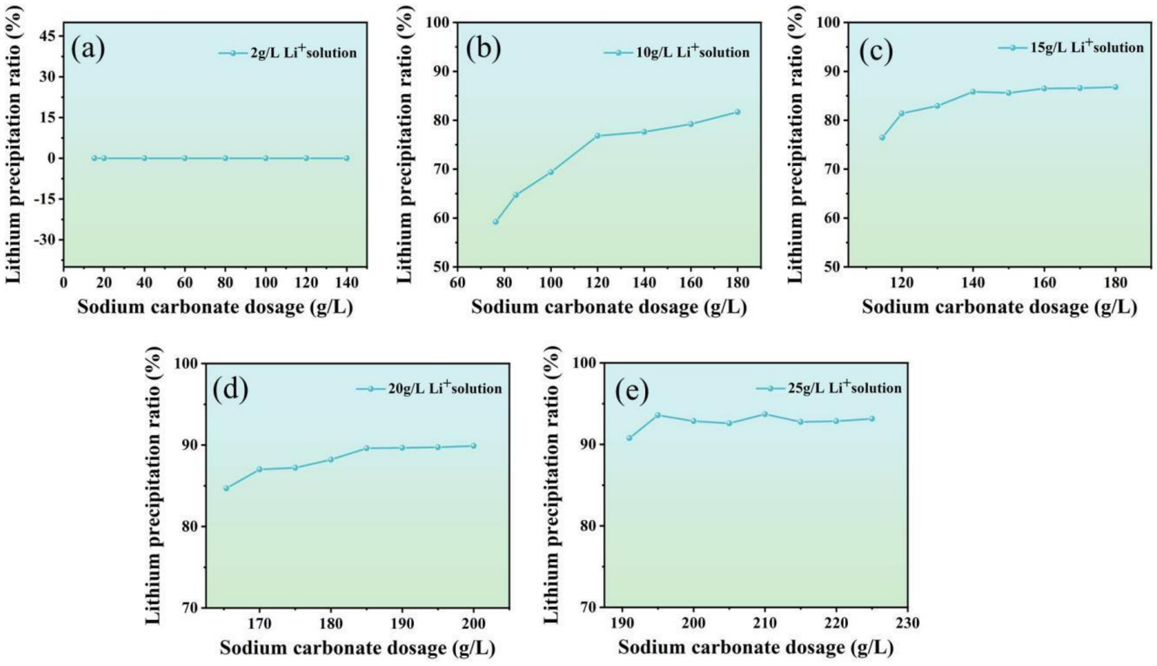

Figure S2. The influence of Na_2CO_3 addition on the Li precipitation rate at different Li concentrations: (a) 2g/L, (b) 10g/L, (c) 15g/L, (d) 20g/L, (e) 25g/L.

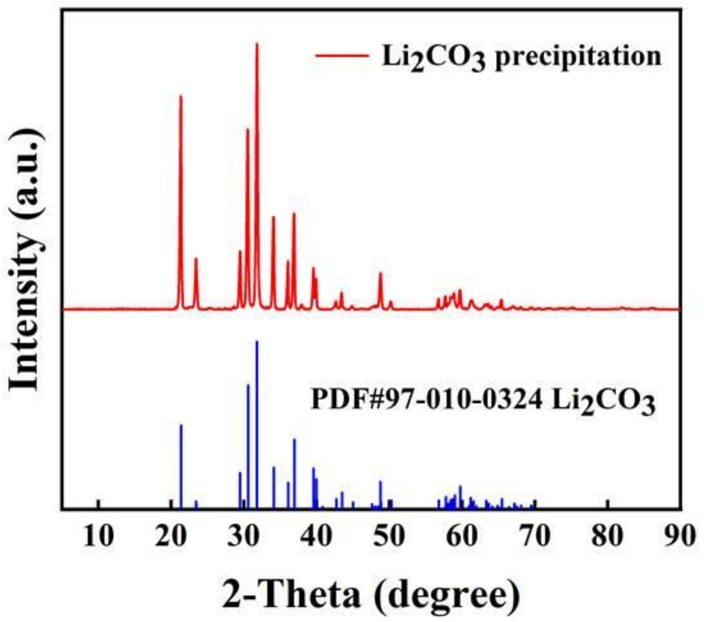

Figure S3. XRD pattern of the obtained Li_2CO_3 precipitation recovered from SLFP.

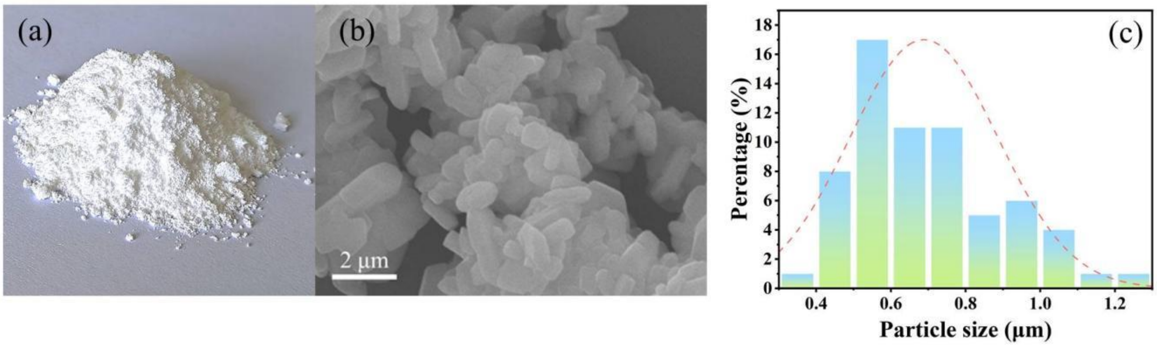

Figure S4. The photo (a), SEM image (b) and particle size distribution (c) of the recovered Li_2CO_3 precipitation recovered from SLFP.

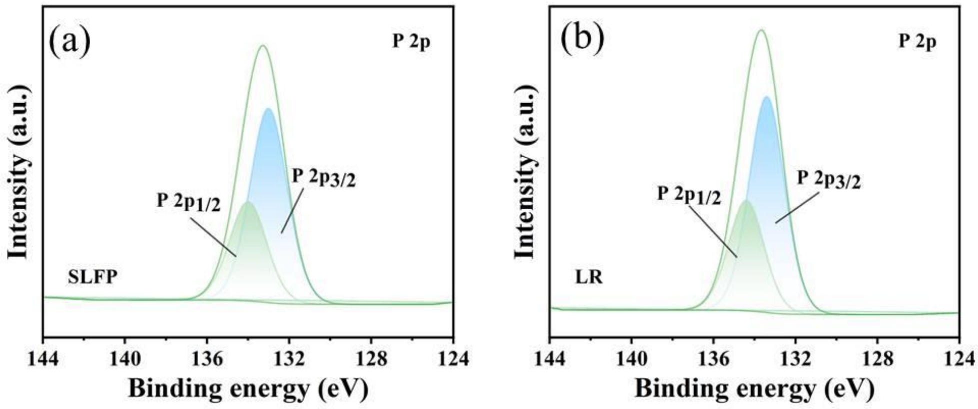

Figure S5. XPS spectra of P 2p: (a) SLFP, (b) LR.

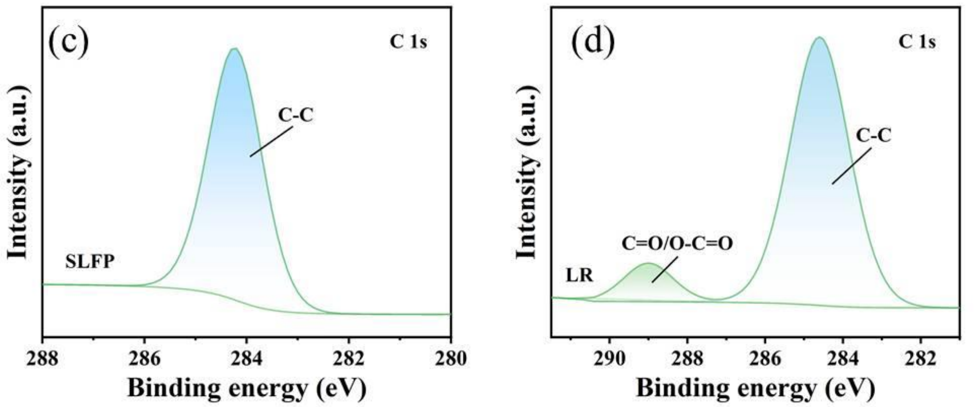

Figure S6. XPS spectra of C 1s: (c) SLFP, (d) LR.

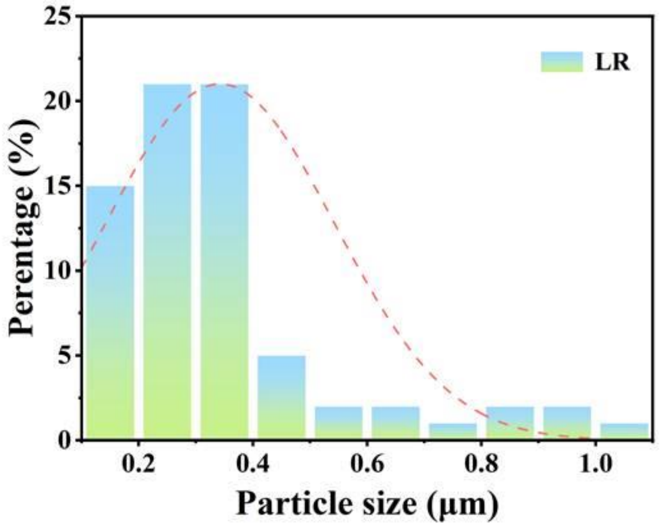

Figure S7. The particle size distribution of LR.

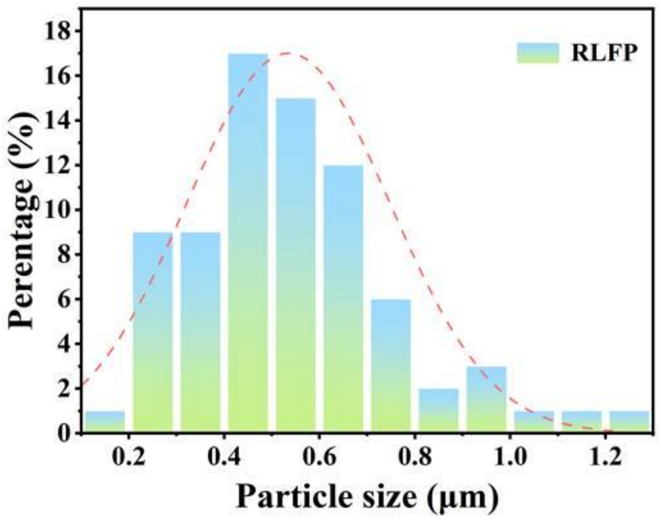

Figure S8. The particle size distribution of RLFP.

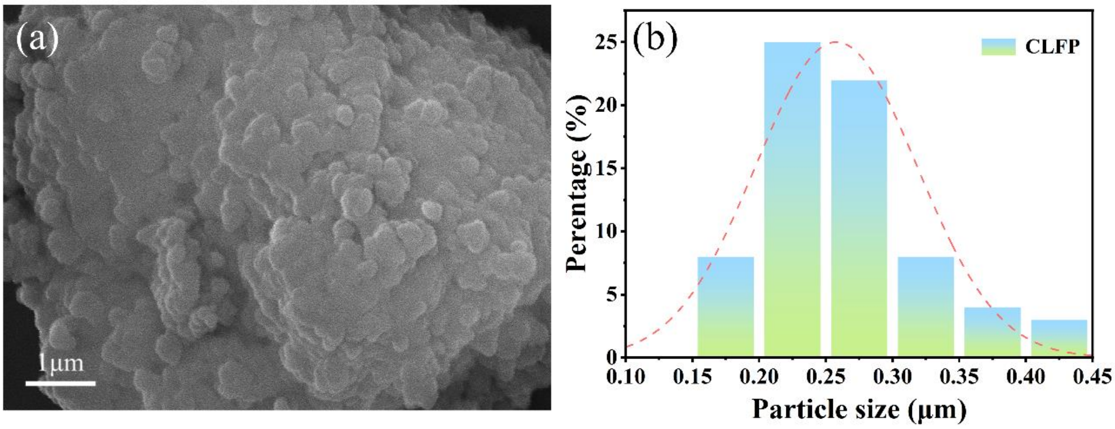

Figure S9. The SEM image (a) and the particle size distribution (b) of CLFP.

Table S1. Comparison of the relevant literature on the selective leaching of LFP cathode materials

Ref.	Leaching conditions	Leaching rate	H_2SO_4/Li molar ratio	Products	Advantages & Limitations
[47]	0.3 M H_2SO_4<br>60 °C, 120 min<br>H_2O_2/Li=2.07	Li 96.85%<br>Fe 0.027%	0.57	Li_3PO_4<br>$FePO_4$	First proposed the stoichiometric acid selective leaching. Long leaching time and no closed-loop regeneration.
[48]	80 mM $FeSO_4$<br>H_2O_2/Li=3.15<br>20 g/L, 40 °C<br>30 min	Li 99.9%<br>Fe<0.01%	phosphoric acid instead of sulfuric acid	Li_2CO_3<br>$FePO_4$<br>Regenerated LFP	Fenton oxidation is adopted, and the leaching speed is fast. The consumption of H_2O_2 is large and a catalyst is needed.
[49]	0.3 M H_2SO_4<br>25 °C, 90 min<br>H_2O_2/Li=1.03	Li 99.99%<br>Fe 0.02%	0.52	Li_2CO_3<br>$FePO_4$	Leaching at room temperature with a low amount of acid. It does not involve material regenerating.
[50]	0.25 M H_2SO_4<br>3% v/v H_2O_2<br>25 °C, 60 min	Li 90±3%<br>Fe 2±1%	0.79	Li_2CO_3<br>$FePO_4$	Low-temperature operation, low cost. The leaching rate of Li is low and the material is not regenerated.
[51]	2.0 M H_2SO_4<br>0.02% v/v H_2O_2<br>30.02 °C, 240 min, 96.88 g/L	Li>2400mg/L	3.26	Leachate	Optimize the parameters through orthogonal experiments. The leaching time is too long, the acid concentration is high.
[52]	2.0 M H_2SO_4<br>H_2SO_4/ H_2O_2 (v/v)=4, 60 °C<br>80 min, 30 g/L	Fe>90%<br>P>85%	10.5	$FePO_4$<br>Regenerated LFP	The synchronous recovery rate of Fe/P is high. Complex post-processing is required (such as pH adjustment).
This work	0.9 M H_2SO_4<br>45 °C, 10 min<br>H_2O_2/Li=2.1	Li 98.72%<br>Fe 0.219%	0.57	Li_2CO_3<br>$FePO_4$<br>Regenerated LFP	The fastest leaching time, closed-loop regeneration, economically feasible.

Table S2. Reference sources for parameter costs

Materials	Reference
Chemicals	Market quotes from Sinopharm Chemical Reagent Co., Ltd. (2024).
Waste LFP battery black powder	Industry reports (https://ldcfl.mysteel.com/).
Electricity	The general industrial and commercial electricity prices announced in Shandong Province, China, are calculated based on the normal standard as the prices fluctuate with peak and off-peak periods.

Water	The non-residential water prices announced in Shandong Province, China, include water resource tax, sewage treatment fees, etc.
N_2	Supplier quotations for industrial-grade nitrogen gas.

Calculation method of electricity consumption: Electricity usage for each step (e.g., leaching, calcination) was calculated by multiplying the equipment's power (kW) by its operation time (hours). (The rated power of the equipment is based on the equipment configuration in the laboratory.)

Table S3. Cost and net product value for one batch experiment

Categories	Cost ($)
Chemicals and spent LFP battery black powder, etc.	-2.0675
Electricity	-0.5580
Resynthesized LFP's value	4.4550
Product's value ($FePO_4$)	0.0690
Total	1.8985
